# Influenza-related Death Rates for Pregnant Women

**DOI:** 10.3201/eid1211.061071

**Published:** 2006-11

**Authors:** Philip Mortimer

**Affiliations:** * Health Protection Agency, London, United Kingdom

**Keywords:** Influenza, mortality, pregnancy, death rates, pregnant women, expecting women, pregnant women

To the Editor: Articles about influenza in the January2006issue of Emerging Infectious Diseases discussed a pandemic possibly as profound in its effect as the 1918–19 pandemic, when attack rates were >20% worldwide and death rates were 1%–2%. Then, as when subsequent virus antigenic shifts have occurred, all age groups were affected. Governments are now preparing contingency plans against the effects of an expected further antigenic shift.

However, insufficient consideration may have been given to how, in the absence of effective prophylaxis against a novel strain of influenza virus, to avoid deaths on the scale seen in the fall and winter of 1918–19. In particular, the vulnerability of pregnant women and their offspring appears to have been forgotten. Bland reported on pregnant influenza patients in Philadelphia and elsewhere in the fall of 1918; of 337, 155 died (*1*). Harris obtained by questionnaire from obstetricians medical histories of 1,350 pregnant patients in Maryland and in 4 large US cities (*2*). Pneumonia developed in half (678) of these patients and 365 died. Death rates from pneumonia were >40% for every month of pregnancy; fetal loss was >40% in all months but the fifth (37%).

According to a contemporaneous report from England, the influenza death rate for pregnant women was 25.4% (*3*). These inquiries into pregnancy must have been biased toward severe cases, but the influenza pandemic in 1918–19 may nevertheless have decreased live births in England and Wales, which reached new lows in the first half of 1919 (*4*). A controlled American study during 1975–1979 has since confirmed that pregnant women are at risk for influenza even in interpandemic years (*5*).

After an interpandemic interval >35 years, any antigenic shift may again seriously affect young adults, including many pregnant women. Preparedness should therefore ensure the availability of timely and comprehensive management of influenza during pregnancy.
